# An Intractable Molar

**Published:** 1880-01

**Authors:** P. K. Stoddard

**Affiliations:** Plattsburgh, N. Y.


### AN INTRACTABLE MOLAR.

BY P. K. STODDARD, M. D., PLATTSBURGH, N. Y.

Some time ago I extracted with a turn key an inferior wisdom tooth, which, when loosened, slipped from the instrument and disappeared. On looking into the socket, which appeared unusually large and deep, I saw nothing, but by exploring it with an instrument found the tooth drawn deeply into the tissues in a direction downward and backward. Seizing it with a pair of slender forceps I dissected it out and found a small portion of the alveoli attached, into this was inserted the muscular fibres which had caused the retraction. I regard such an accident as very uncommon, and mention it because some dentist was prosecuted for a similar accident. In that case the tooth was not found till after it had caused great suffering, inflammation and abscess.

The accident in both cases was probably unavoidable and justifiable; not to find and remove it may have been culpable malpractice. —*Medical and Surgical Brief, N. Y.*

